# Delineation and authentication of ferroptosis genes in ventilator-induced lung injury

**DOI:** 10.1186/s12920-024-01804-y

**Published:** 2024-01-23

**Authors:** Enhao Huang, Hanghang Han, Ke Qin, Xueke Du

**Affiliations:** Department of Anesthesiology, The Second Affiliated Hospital of Guangxi Medical University, Guangxi Zhuang Autonomous Region, Nanning, 530007 China

**Keywords:** Ventilator-induced lung injury, Ferroptosis, Biomarkers, Bioinformatics

## Abstract

**Background:**

Mechanical ventilation, a critical support strategy for individuals enduring severe respiratory failure and general anesthesia, paradoxically engenders ventilator-induced lung injury (VILI). Ferrostatin-1 mitigates lung injury via ferroptosis inhibition, yet the specific ferroptosis genes contributing significantly to VILI remain obscure.

**Methods:**

Leveraging the Gene Expression Omnibus database, we acquired VILI-associated datasets and identified differentially expressed genes (DEGs). To identify the hub genes, we constructed a protein–protein interaction network and used three parameters from CytoHubba. Consequently, we identified hub genes and ferroptosis genes as ferroptosis hub genes for VILI (VFHGs). We conducted enrichment analysis and established receiver operating characteristic (ROC) curves for VFHGs. Subsequently, to confirm the correctness of the VFHGs, control group mice and VILI mouse models, as well as external dataset validation, were established. For further research, a gene-miRNA network was established. Finally, the CIBERSORT algorithm was used to fill the gap in the immune infiltration changes in the lung during VILI.

**Results:**

We identified 64 DEGs and 4 VFHGs (*Il6*,*Ptgs2*,*Hmox1* and *Atf3*) closely related to ferroptosis. ROC curves demonstrated the excellent diagnostic performance of VFHGs in VILI. PCR and external dataset validation of the VILI model demonstrated the accuracy of VFHGs. Subsequently, the gene-miRNA network was successfully established. Ultimately, an Immune cell infiltration analysis associated with VILI was generated.

**Conclusions:**

The results emphasize the importance of 4 VFHGs and their involvement in ferroptosis in VILI, confirming their potential as diagnostic biomarkers for VILI.

**Supplementary Information:**

The online version contains supplementary material available at 10.1186/s12920-024-01804-y.

## Background

Ventilator-induced lung injury (VILI) signifies an acute pulmonary impairment or exacerbation thereof, engendered by the clinical deployment of mechanical ventilation [1]. Predominantly, patients bearing preexisting pulmonary afflictions exhibit an amplified susceptibility to VILI exacerbation [2]. Several elements, encompassing air pressure trauma and cochlear and pulmonary cell electrophysiology, are postulated as potential VILI provocateurs [3].

Ferroptosis represents a form of regulated cellular demise, distinguished by intracellular iron overaccumulation, culminating in an escalation of lipid peroxides. Evidence underscores a robust correlation between pulmonary diseases and ferroptosis progression, as exemplified by conditions such as chronic obstructive pulmonary disease [4, 5], asthma [6] and ALI [7–9]. Ling and colleagues corroborated the efficacy of ferrostatin-1 in mitigating VILI by impeding ferroptosis, underscoring the occurrence and aggravation of ferroptosis during VILI evolution [10], although the genetic mechanism remains elusive.

The bioinformatics analysis of ferroptosis genes in VILI remains unexplored. In this study, we conducted a comprehensive investigation by analyzing lung tissue microarray datasets from seven distinct VILI samples obtained from the GEO database. Through this analysis, we identified differentially expressed genes (DEGs) in pulmonary cells associated with VILI. These DEGs were then utilized to construct a relevant protein–protein interaction (PPI) network. Furthermore, we applied three topology algorithms using the CytoHubba plugin in Cytoscape software to identify hub genes, resulting in the identification of VILI ferroptosis hub genes (VFHGs). Enrichment analysis was performed to elucidate the potential functions of VFHGs and the accuracy of our selection was validated through various approaches, including the establishment of VILI mouse models for qPCR analysis and external dataset validation. To further investigate the VFHGs, we constructed a gene-miRNA network to explore their potential functional roles. Finally, considering the potential link between ferroptosis and immunity, we analyzed the immune infiltration of VILI using the CIBERSORT algorithm. Our findings shed light on the involvement of ferroptosis genes in the pathogenesis of VILI, providing a basis for understanding the underlying pathological mechanisms.

## Method

### Data acquisition

The datasets GSE9314, GSE9368, GSE11434, GSE29920, GSE2368, GSE121550 and GSE86229 were retrieved from the GEO database (https://www.ncbi.nlm.nih.gov/geo/). The first six gene chip datasets were utilized for the screening of VILI ferroptosis hub genes (VFHGs), while the last gene chip dataset served as an external validation set for the identified VFHGs. Ferroptosis-relevant genes were retrieved from the Ferrdb database (http://www.zhounan.org/ferrdb/legacy/operations/) [11]. The sample inclusion criterion dictated the utilization of free ventilated mice as the control group and mechanically ventilated mice as the experimental cohort (Table 1).

**Table 1:** Gene chip data

Accession	Platform	Sample	Control Group	VILI Group
GSE9314	GPL1261	Lung tissue	4	4
GSE9368	GPL1261	Lung tissue	3	3
GSE11434	GPL1261	Lung tissue	5	5
GSE29920	GPL6885	Lung tissue	3	5
GSE2368	GPL81	Lung tissue	2	2
GSE121550 male	GPL16570	Lung tissue	3	3
GSE121550 female	GPL16570	Lung tissue	3	3
GSE86229	GPL6246	Lung tissue	5	10

### Differential gene expression analysis

Microarray probes were converted into gene symbols using R (version 4.2.1). In cases where multiple probes corresponded to a single gene ID, the average value was computed for subsequent analysis. In addressing the batch effects arising from the utilization of different microarray platforms, the Surrogate Variable Analysis (SVA) package was employed for batch effect correction [12]. SVA provides a powerful and flexible framework for the identification and removal of hidden sources of variation, such as batch effects, while preserving the biological variability of the integrated dataset. The SVA package effectively models and estimates batch effects, allowing for the correction of systematic biases and ensuring that the integrated dataset accurately reflects the underlying biological signals across the combined datasets.

In summary, the integration of the six datasets into a single integrated dataset involved a robust and comprehensive approach aimed at mitigating technical variations and batch effects, thereby ensuring the generation of a unified dataset that accurately captures the biological signals of interest across the combined datasets [13–15].

Principal component analysis (PCA) was conducted to identify significant dimensions of variation in each dataset. Quality control was performed using boxplot analysis. Statistical significance was determined by considering Adj.P.Val < 0.05 and |logFC|> 1. Volcano plots were generated using the ggplot2 package in R, while gene heatmaps were constructed using the ComplexHeatmap package.

### Hub gene and VFHG acquisition

DEGs were subjected to PPI analysis using the String database (https://cn.string-db.org/). The analysis was performed with a species restriction to *Mus musculus* and a minimal correlation coefficient threshold of 0.4. The resulting interaction data from the String database were imported into Cytoscape software (version 3.9.1), which included the cytoHubba plugin. The cytoHubba plugin screened the top 10 genes based on three parameters [16, 17]: maximal clique centrality (MCC), maximum neighborhood component (MNC) and degree. The intersection of the hub genes obtained using the three parameters and the set of ferroptosis genes yielded the final selection of VFHGs.

### GO, KEGG analysis and visualization

The clusterProfiler package in R software was utilized for performing Gene Ontology (GO) and Kyoto Encyclopedia of Genes and Genomes (KEGG) enrichment analysis [18–20] of the molecular list after ID conversion. GO encompasses biological processes (BP), cellular components (CC) and molecular functions (MF) to describe gene functions and interactions. The KEGG Pathway Database is a comprehensive resource used for annotating pathways. Subsequently, the ggplot2 package was employed to visualize the results of the enrichment analysis.

### ROC curve

To comprehensively assess the diagnostic performance of VFHGs, ROC curve analysis was conducted utilizing the pROC package in R [21]. The ROC curve provides a graphical representation of the trade-off between sensitivity and specificity across various cutoff points, allowing for the evaluation of the discriminatory power of the VFHGs [22]. Specifically, the pROC package enabled the calculation of the area under the ROC curve (AUC), a summary statistic that quantifies the overall discriminatory capacity of the VFHGs as a diagnostic biomarker. The ROC curve generated through this analysis provides a visual depiction of the performance characteristics of the VFHGs, thus allowing for a comprehensive assessment of their utility in diagnostic applications.

### Animal model

Eight immunocompetent male C57BL / 6 J mice, aged 6 ~ 8 weeks, were provided by the Experimental Animal Center of Guangxi Medical University (animal certificate number: SYXK (Gui) 2014–0003). The protocol adhered to the ethical requirements of experimental animal welfare and obtained the consent of the Second Affiliated Hospital of Guangxi Medical University Ethical Review Committee. The animal study was conducted following ARRIVE guidelines (https://arriveguidelines.org).

### HE staining and TEM

Eight male C57BL/6 J mice were randomly assigned to control and VILI groups, with 4 mice in each group. The mice in the control group were intubated but did not undergo mechanical ventilation; the VILI group was mechanically ventilated with a tidal volume of 20 mL/kg for a period of four hours. Prior to all procedures, the mice were intraperitoneally administered 1% pentobarbital at 50 mg / kg, with gentle insertion of the tracheal tube. The mice in both the control and VILI groups were under 1% pentobarbital anesthesia after tracheal intubation. Four hours later, following cardiac puncture for blood collection, the anesthetized mice were euthanized by removal of the lungs upon cessation of breathing. The upper and lower lobes of the left lung from both mouse groups were employed for electron microscopy and HE staining to verify model success, with the lower lobe of the right lung utilized for RNA extraction for qRT-PCR treatment. Detailed methodologies for transmission electron microscopy and HE staining are presented in Supplementary Table 1.

### RNA extraction and qPCR

Lung tissues from each mouse were procured for quantitative PCR (qPCR) to validate the expression of VFHGs. As per the protocol furnished by the reagent instructions, total RNA was extracted from mouse lung tissue using TRIzol reagent. After extraction, the concentration and purity of each RNA group were ascertained, with the benchmark being an A260/A280 ratio of 1.00–2.00. Each RNA sample (1 μg) was reverse transcribed into cDNA via the PrimeScript™ RT Master Mix kit (RR036A, TaKaRa), which then served as a template for PCR amplification. The primers were synthesized by Shanghai Sangon Biotechnology Co, Ltd. The utilized primer sequences are illustrated in Table 2. The relative expression level of the target gene was calculated following the 2^−ΔΔCt^ methodology.

**Table 2:** Specific primer of sequences used in PCR

Gene	Primer sequences(5′-3′)
*Hmox1*	F:CTAGCCTGGTGCAAGATACTG
	R:GGCATAAATTCCCACTGCCAC
*Atf3*	F:TTACCGTCAACAACAGACCCC
	R:CTCTCCAGTTTCTCTGACTCTTTC
*Ptgs2*	F:TGGGGGAAGAAATGTGCCAA
	R:CAGCCATTTCCTTCTCTCCTGT
*Il6*	F:CTCCCAACAGACCTGTCTATAC
	R:CCATTGCACAACTCTTTTCTCA
*Il1b*	F:CACTACAGGCTCCGAGATGAACAAC
	R:TGTCGTTGCTTGGTTCTCCTTGTAC
*Tnfα*	F:ATGTCTCAGCCTCTTCTCATTC
	R:GCTTGTCACTCGAATTTTGAGA

### Gene-mRNA interaction network

The comprehensive interaction network between genes and miRNAs pertaining to VFHGs was formulated employing the Network Analyst web portal (https://www.networkanalyst.ca/) [5]. The process involved choosing comprehensive, experimentally validated miRNA-gene interaction data collated from TarBase while constraining the search species to '*Mus musculus* (mouse)' and ID type to ‘Official Gene Symbol’.

### Immune cell infiltration analysis

The immune microenvironment typically consists of immune cells, inflammatory cells, fibroblasts and chemokines. Immune cell infiltration analysis plays a critical guiding role in predicting disease progression and treatment response [23]. The CIBERSORT algorithm employs linear support vector regression to estimate the proportions of immune cells in samples using RNA sequencing data [24]. In our study, we utilized the CIBERSORT algorithm in R software to calculate the differences in immune cell composition between VILI mice and control mice in the dataset and visualized the results using the ggpubr package.

### Statistical analysis

The pertinent data underwent statistical scrutiny employing R software in tandem with SPSS 25 (IBM, USA). A T test was leveraged for the statistical exploration of normally distributed data and disparities accompanied by a bilateral *P* value of < 0.05 were deemed statistically significant.

## Results

### Acquisition of DEGs

**Fig. 1:**
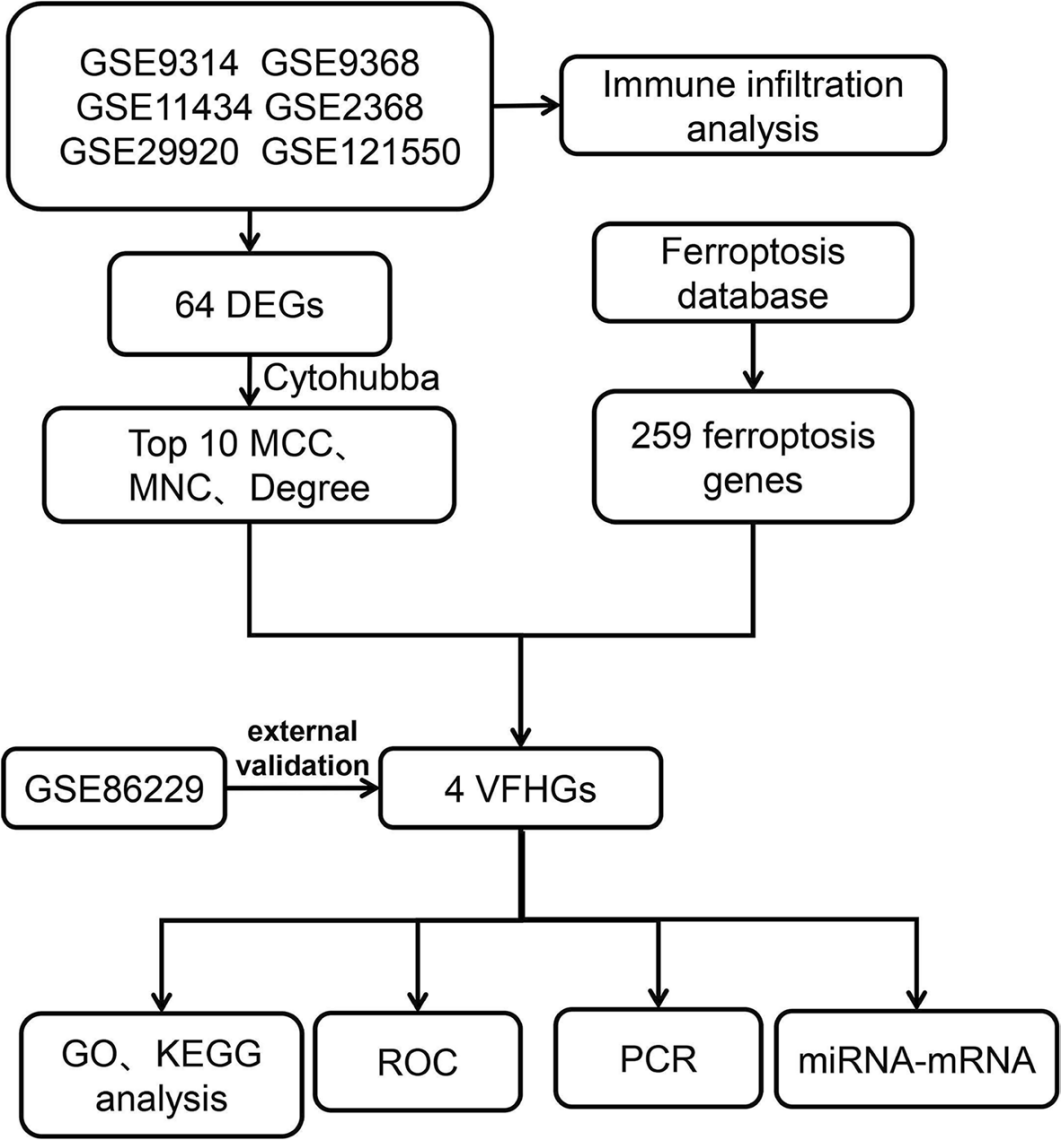
Study schematic diagram

The flowchart of the study is presented in Fig. 1. The data were divided into two groups: the control (CON) group and the VILI group. Subsequently, PCA and boxplot analysis were performed on the data after applying quality control measures and standardizing the original data [25]. The PCA results revealed significant differences between the CON and VILI groups (Supplementary Fig. 1A). The boxplot demonstrated the acceptability of normalizing the dataset after correcting for batch effects using the combat package (Supplementary Fig. 1B).

**Fig. 2:**
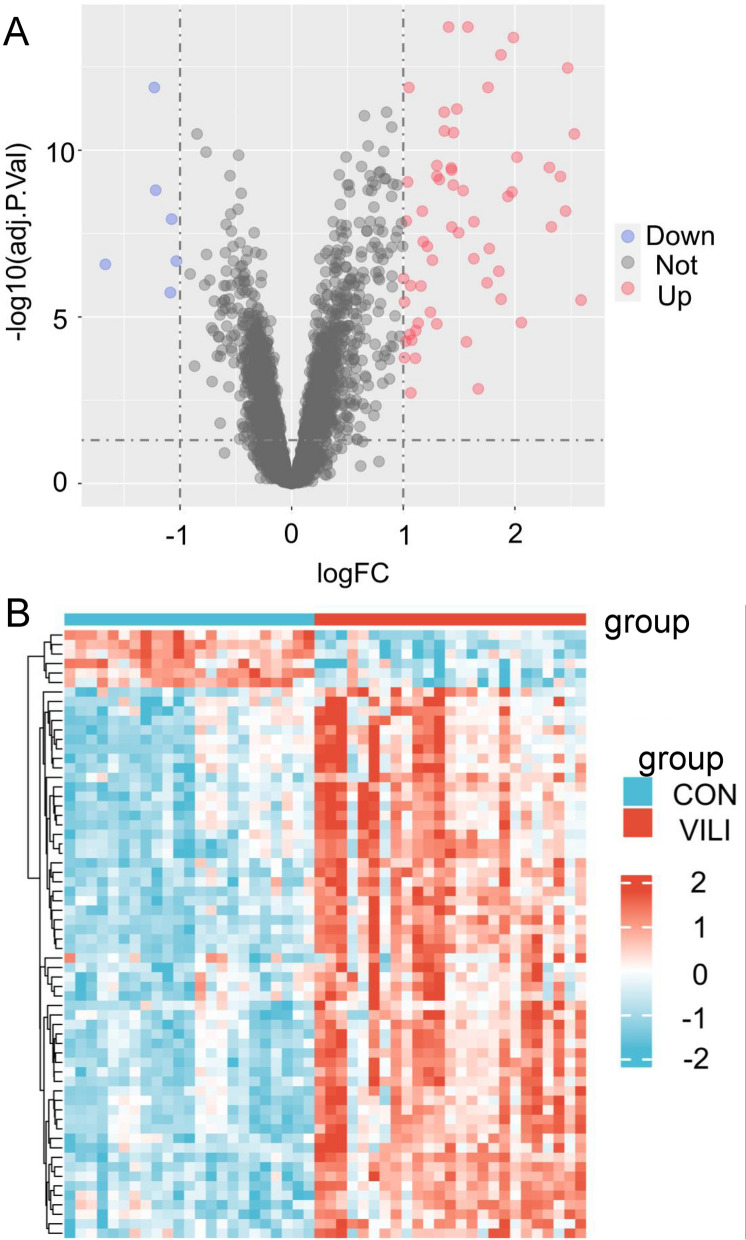
Acquisition of DEGs in ventilator-induced lung injury. **A** Volcanic map of DEGs: red represent up-regulated genes and blue represents downregulated genes. **B** Gene expression heatmap of 64 DEGs: red represents high gene expression and blue represents low gene expression

DEG analysis was conducted using the Limma software package, leading to the identification of 64 DEGs, including 58 upregulated genes and 6 downregulated genes. In order to present DEGs more intuitively, volcano plots and gene heatmaps were generated using the ggplot2 and ComplexHeatmap packages, respectively (Fig. 2A, B).

### PPI analysis and VFHGs acquisition

**Fig. 3:**
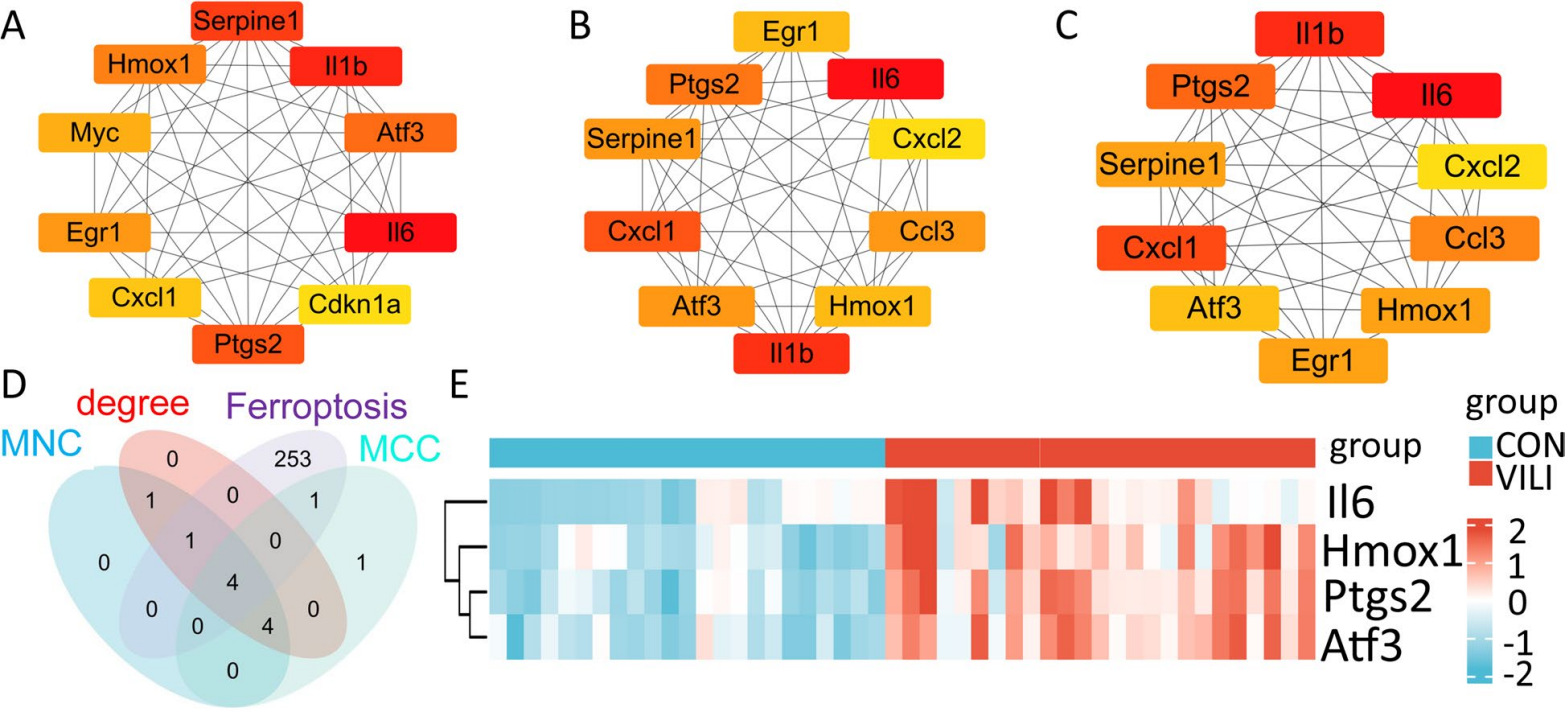
Identification of hub genes and identification of VFHGs. **A**-**C** Sixty-four differentially expressed genes were screened using MCC, degree and MNC topology algorithms to identify the top 10 hub genes. **D** The intersection genes of the top 10 hub genes and ferroptosis genes screened by the MCC, MNC and degree topology algorithms are the VFHGs. **E** Gene expression heatmap of VFHGs: red represents high gene expression and blue represents low gene expression

PPI relationships among the DEGs were established using the STRING database. The top 10 genes were selected using three topology algorithms (MCC, Degree and MNC) from the cytoHubba plugin in Cytoscape (Fig. 3A-C). These top genes were then combined with the ferroptosis genes to obtain VFHGs (Fig. 3D). Additionally, a gene heatmap of VFHGs was generated using the ggplot2 package (Fig. 3E).

### GO and KEGG pathway analysis

**Fig. 4:**
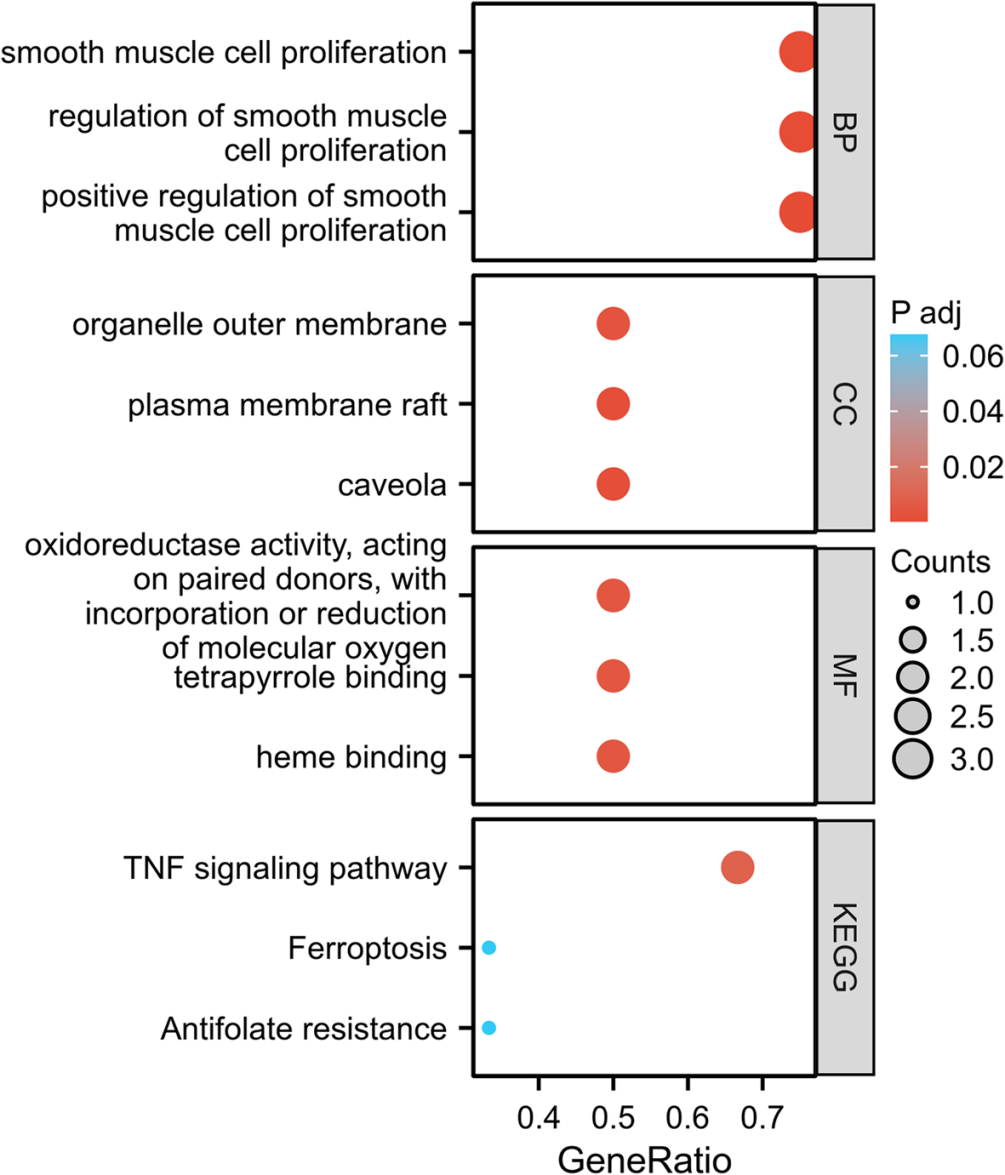
Functional enrichment analysis of DEGs: the y-axis represents GO and KEGG terms; the x-axis represents gene ratio involved in corresponding GO and KEGG terms. The size of circles represents gene numbers, and their color refers to adj.*p*-value

Enrichment analysis was performed on the identified VFHGs and detailed results of BP, CC, MF and KEGG pathways were provided (Supplementary Table 2). The analysis revealed that VFHGs are primarily involved in well-established NF-κB inflammatory pathways, antifolate resistance, ferroptosis and TNF signaling pathways. To visualize these enrichment analysis results, we generated Fig. 4.

### Diagnostic accuracy of VFHGs

**Fig. 5:**
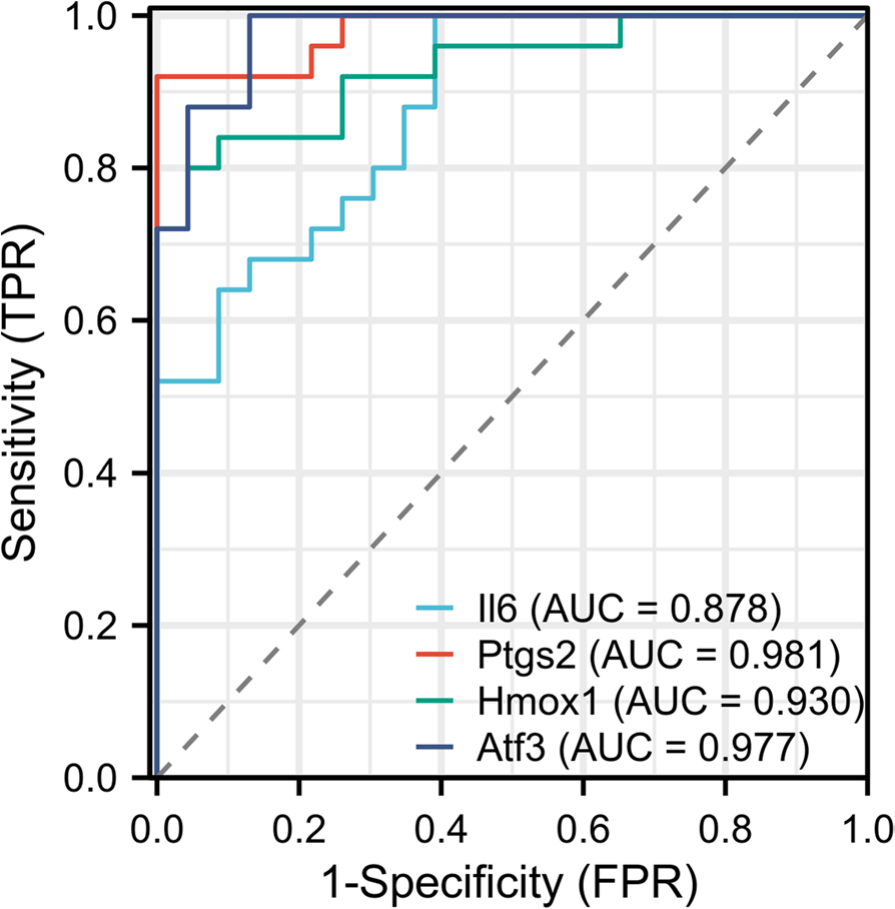
ROC curve of VFHGs: the area under the curve represents the AUC of the gene

To validate the diagnostic accuracy of the selected VFHGs for VILI, we conducted data analysis using the pROC package and visualized the results using ggplot2 (Fig. 5). The results indicated that the AUC for the four VFHGs exceeded 0.85, suggesting excellent diagnostic performance for VILI.

### The successful establishment of VILI model

**Fig. 6:**
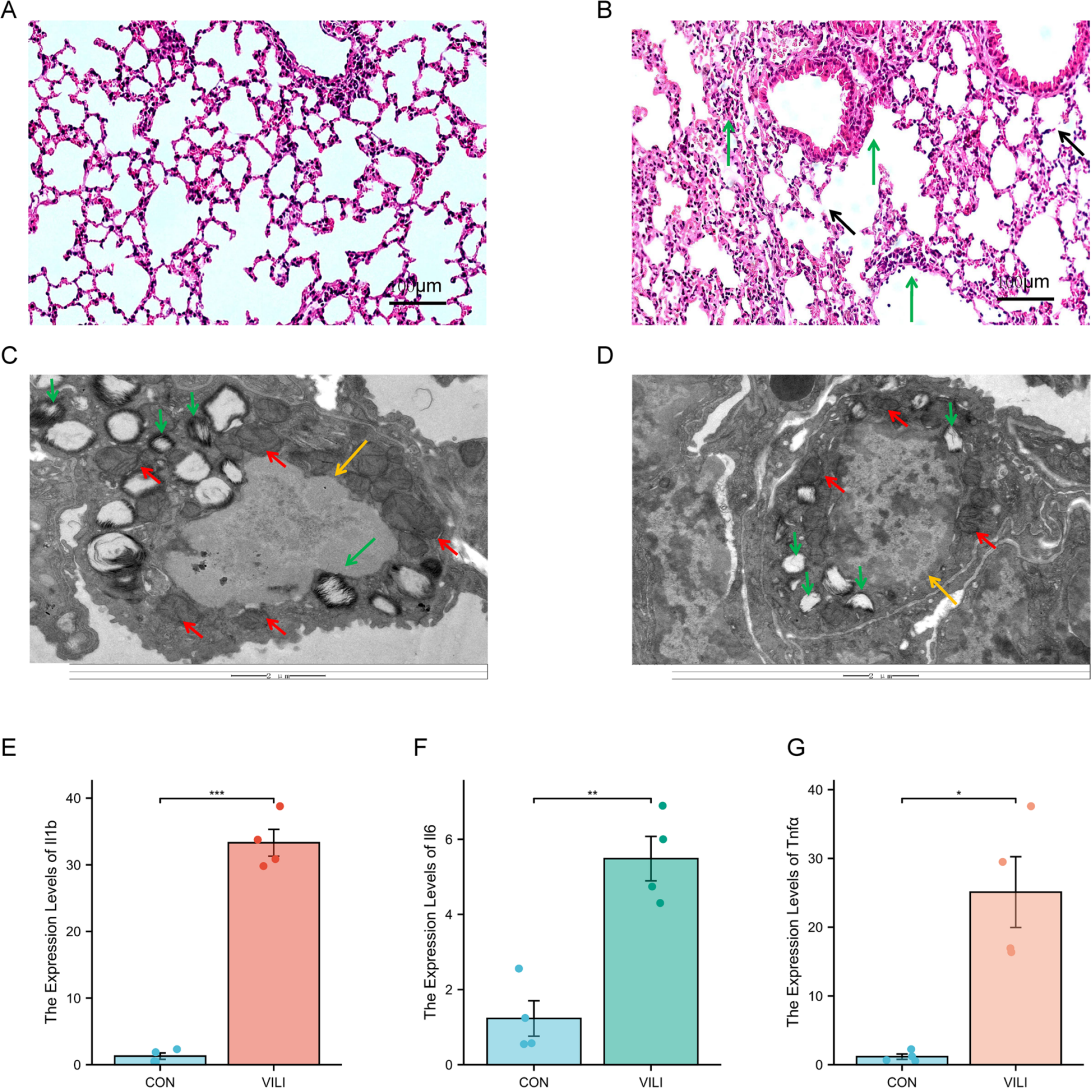
The successful establishment of the VILI model. **A** HE tissue staining of the lungs of spontaneously breathing mice. **B** HE tissue staining of the lungs of VILI mice. **C** Transmission electron microscopy results of the lungs of spontaneously breathing mice. **D** Transmission electron microscopy results of the lungs of VILI mice. **E**–**G** mRNA expression of the inflammatory factors *Il1b, Il6 and Tnfα* in the lungs of CON and VILI mice.*n* = 4. Mean ± SEM, * *P* < 0.05 vs. CON group, **P < 0.01 vs. CON group, ***P < 0.001 vs. CON group

After four hours of mechanical ventilation, histopathological examination using HE staining revealed significant lung damage in the mice (Fig. 6A, B). The CON group exhibited intact alveolar septa without inflammatory cells within the alveolar septa and cavity and no pulmonary exudation. In contrast, the VILI group displayed thickening of the alveolar septa, alveolar wall rupture (indicated by the black arrow), significant infiltration of inflammatory cells in the alveolar septa and cavity and increased pulmonary exudation (indicated by the green arrow).

Transmission electron microscopy examination revealed ultrastructural alterations in type II alveolar epithelial cells of the mice (Fig. 6C, D). The control group exhibited well-defined nuclear boundaries (indicated by the yellow arrow), mitochondria with a distinct ridge and intact capsular structure (indicated by the red arrow) and laminar bodies with uniform texture density (indicated by the green arrow). Conversely, the VILI group showed nuclear membrane blurring (indicated by the yellow arrow), irregular mitochondrial shape, mitochondrial crista rupture (indicated by the red arrow) and vacuolar lamellar bodies (indicated by the green arrow).

Furthermore, the mRNA expression levels of inflammatory factors (*Il1b, Il6 and TNFα*) were significantly increased in the VILI group, confirming the successful establishment of the VILI model (Fig. 6E-G).

### Identification of VFHGs

**Fig. 7:**
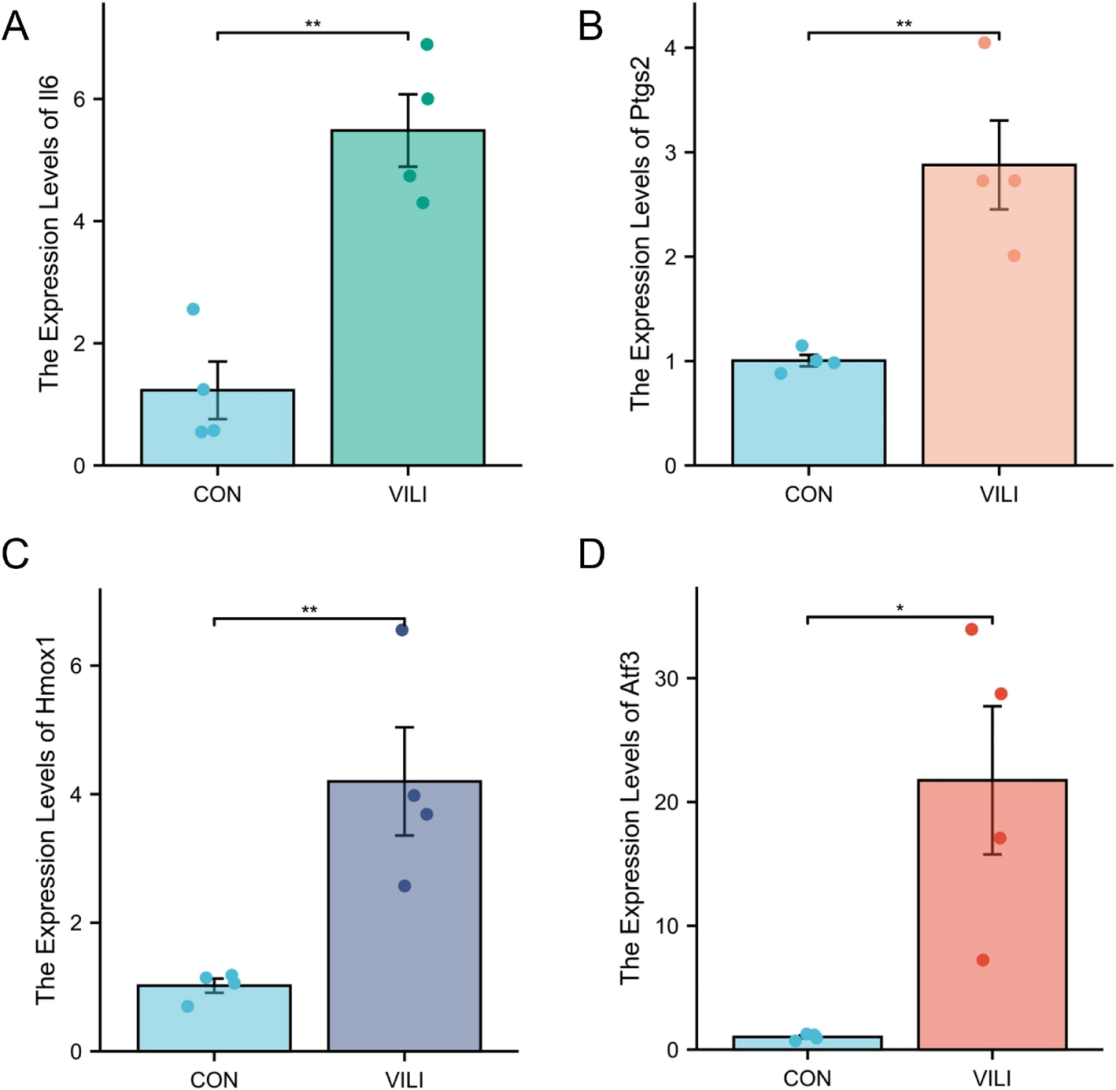
qPCR results showing the expression levels of VILI ferroptosis hub genes in the CON group and VILI group. **A**-**D** *Il6, Ptgs2, Hmox1 and Atf3* mRNA expression in the lungs of CON and VILI mice.*n* = 4. Mean ± SEM, * *P* < 0.05 vs. CON group, **P < 0.01 vs. CON group

The mRNA expression levels of *Il6,Ptgs2,Hmox1 and Atf3* in the VILI group displayed significant upregulation (*p* < 0.05) relative to those in the CON group, a finding in accordance with prior screening and validation results (Fig. 7A-D). This also provides evidence of the correctness of the selected VFHGs from an animal experimental perspective.

### External validation of VFHGs

**Fig. 8:**
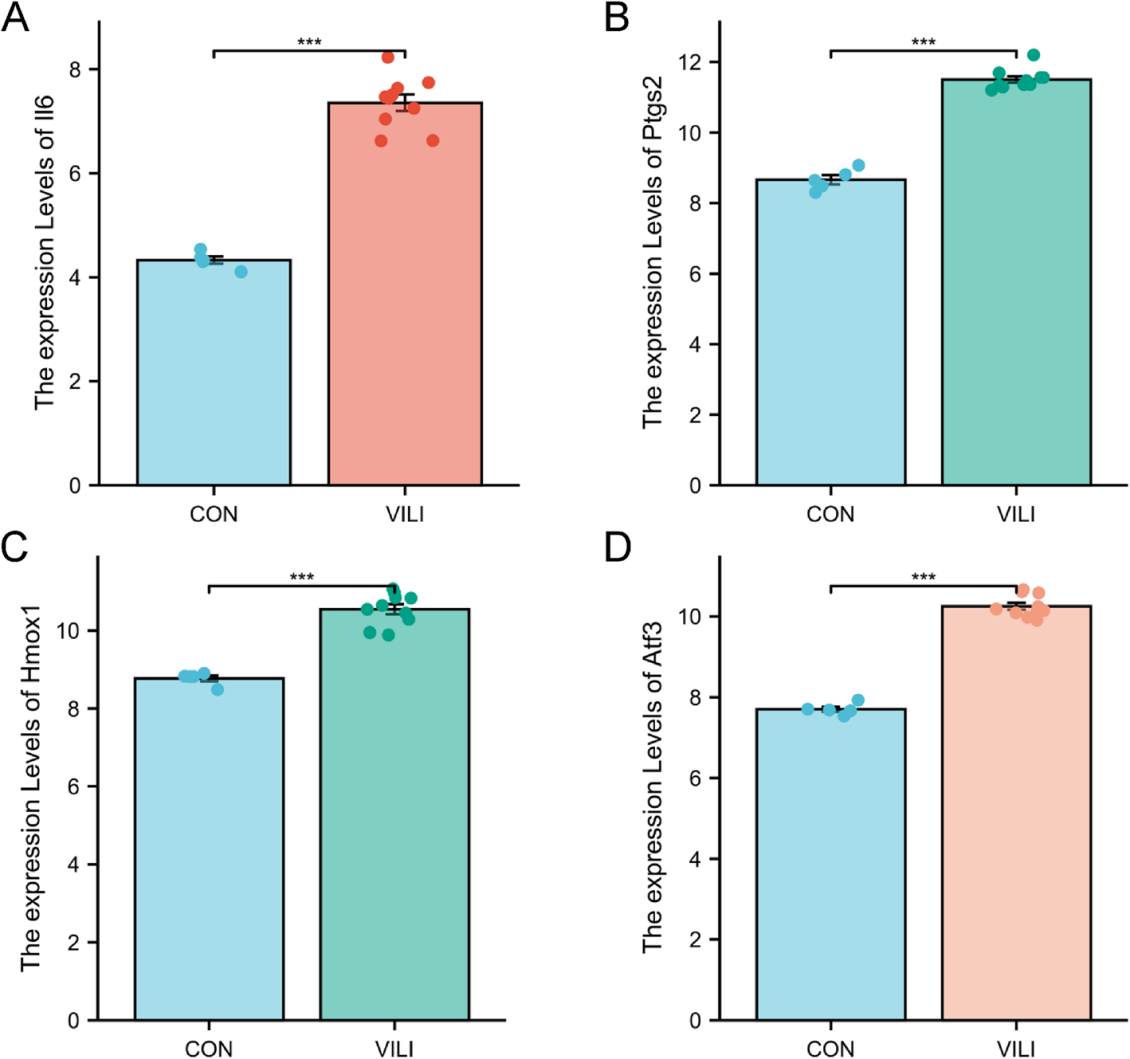
Expression of *Il6,Ptgs2,Hmox1 and Atf3* in the GSE86229 dataset. **A**-**D** *Il6, Ptgs2, Hmox1 and Atf3* expression in the lungs of CON and VILI mice.*n* = 5–10. Mean ± SEM, * *P* < 0.05 vs. CON group, **P < 0.01 vs. CON group, ***P < 0.001 vs. CON group

To further assess the accuracy of the four VFHGs, we performed additional analysis on the GSE86229 dataset. Consistently, the expression levels of the four VFHGs remained elevated in the VILI group compared to the CON group, providing further confirmation of our hypothesis (Fig. 8A-D).

### Gene-miRNA interaction network

**Fig. 9:**
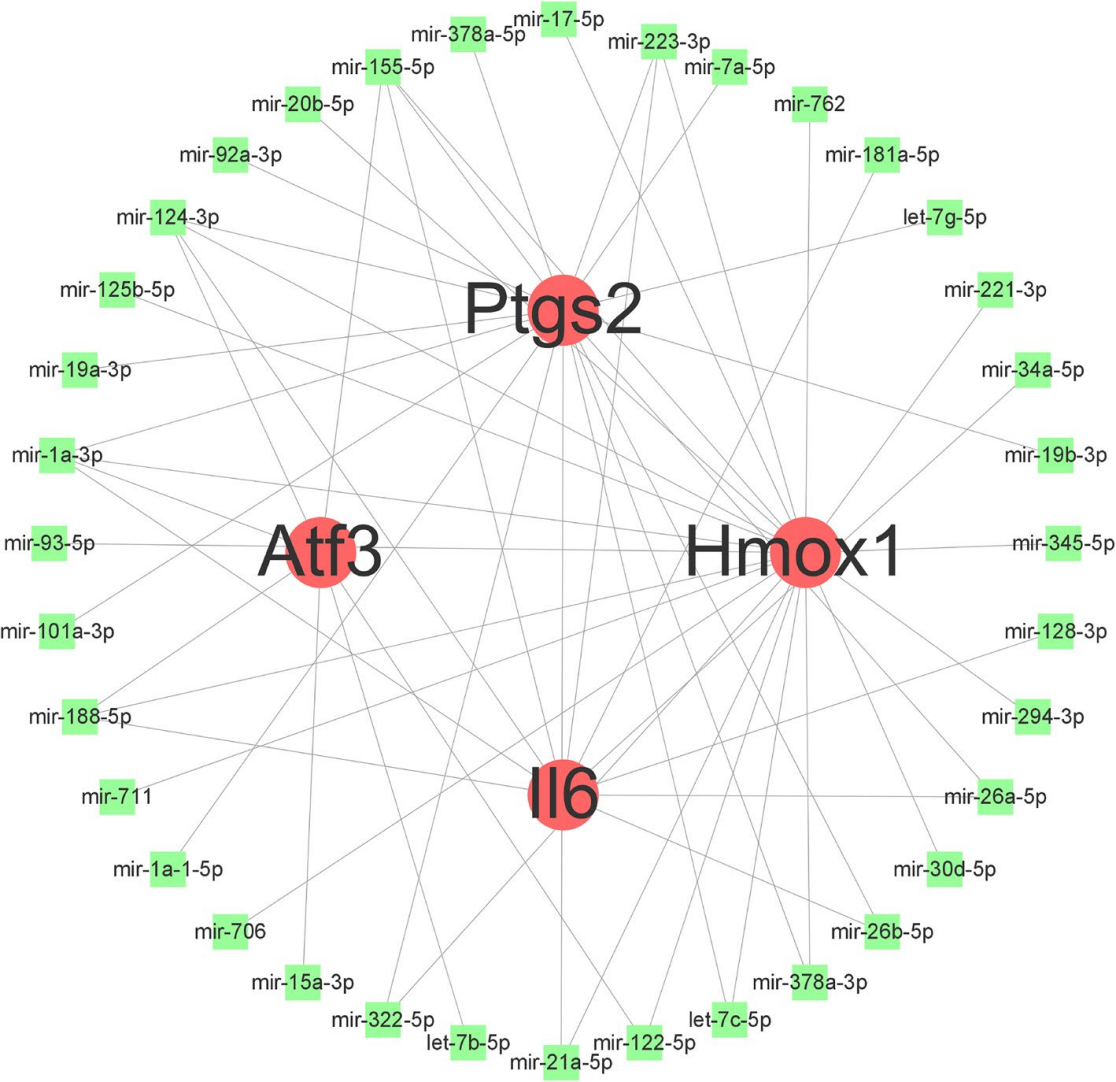
Gene-miRNA network interaction diagram of VILI ferroptosis-related genes: the red circles represent genes, the green squares represent miRNA, and the connecting lines between them represent miRNA-regulated expression

To investigate potential regulatory interactions, we utilized the TarBase v8.0 database and a network analysis website. Through this analysis, we constructed a gene-microRNA interaction network diagram, which consisted of 40 nodes and 56 edges (Fig. 9). In the diagram, genes are represented by red circles, while microRNAs are denoted by green squares. Through this network diagram, we can infer the regulation of VFHGs by relevant miRNAs, which in turn affects the occurrence and development of VILI.

### Immune cell infiltration analysis

**Fig. 10:**
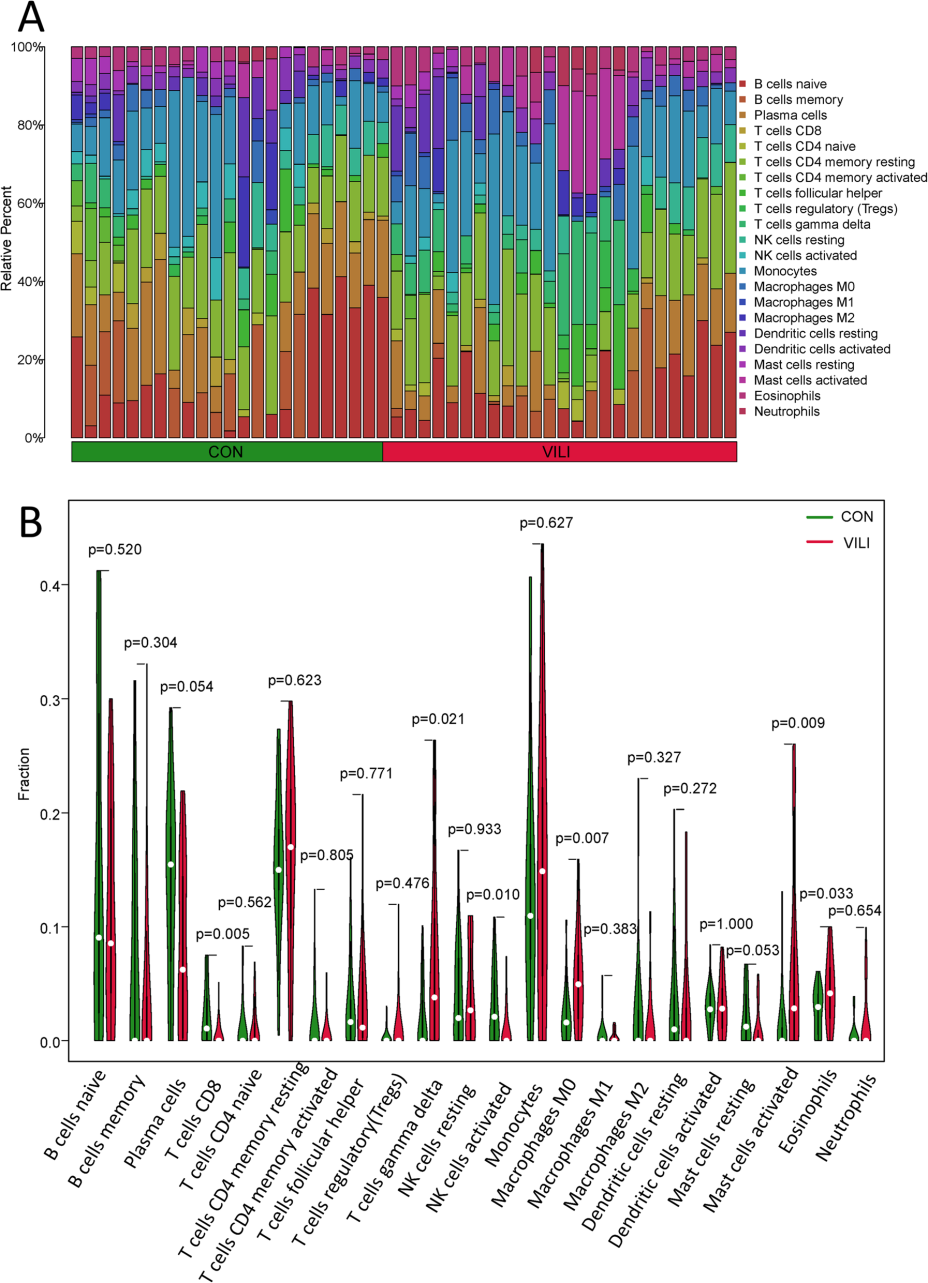
The results of 22 types of immune cell infiltration. **A** The bar plot shows the composition of 22 types of immune cells in the CON and VILI groups. **B** The vioplot plot shows the differences in immune infiltration of 22 immune cells between the CON group and the VILI group

Using the CIBERSORT package to determine changes in the proportions of immune cell types during the VILI process, we obtained the expression levels of 22 different types of immune cells in VILI lung tissue compared to normal lung tissue (Fig. 10A). In comparison to normal lung tissue, there was a decrease in the proportions of CD8 T cells and activated NK cells, while there was an increase in the proportions of gamma delta T cells, M0 Macrophages, activated mast cells and eosinophils (Fig. 10B). This provides strong theoretical support for our subsequent analysis of VILI and the immune cell microenvironment.

## Discussion

VILI has gained significant attention due to its notable clinical implications. Despite extensive research, effective treatments for VILI remain elusive. This study focused on genes related to VILI and ferroptosis, leading to the identification of four key genes closely associated with ferroptosis: Il6, Ptgs2, Hmox1 and Atf3. This discovery provides fresh insights into the molecular mechanisms of VILI, particularly highlighting the role of ferroptosis.

Current paradigms distinguish ferroptosis as an oxidative stress-induced cell death pathway, separate from traditional apoptosis [26]. Prior research has established a strong connection between ferroptosis and lung diseases. However, the mechanisms of ferroptosis and VILI are typically discussed independently [10]. This study bridges this gap by systematically identifying VFHGs: Il6, Ptgs2, Hmox1 and Atf3. These genes were validated for their accuracy and significance using ROC curves and animal models. Enrichment analysis of the VFHGs showed their central roles in oxidative stress and the ferroptosis pathway during VILI initiation and progression. This finding supports the idea that oxidative stress induced by VFHGs is linked to VILI development and underscores the pivotal role of ferroptosis.

Il6 is widely recognized as a significant mediator of inflammation in various lung diseases. In the context of VILI, its role extends beyond mere pro-inflammation, particularly ferroptosis. Studies have suggested that Il6 can foster ferroptosis by modulating cellular iron homeostasis [27]. Thus, within VILI, Il6 may not only spur inflammation but also trigger cell death via ferroptosis, intensifying lung injury.

Ptgs2 primarily governs prostaglandin peroxidase activity and is essential in the pathology of inflammatory signal propagation [28]. Interestingly, suppressing Ptgs2 expression in myocardial infarction models can mitigate cardiomyocyte ferroptosis, reducing tissue damage [29]. However, the intricate relationship between Ptgs2, VILI and ferroptosis warrants further exploration. Gaining insight into Ptgs2's influence on inflammation and ferroptosis in VILI will be instrumental in unveiling novel therapeutic avenues.

In sepsis-induced lung injury, AUF1 modulates NRF2 and Atf3, impacting cellular oxidative stress and iron balance. This modulation resists ferroptosis in lung epithelial cells [30]. Consequently, elevated AUF1 expression during mechanical ventilation may correlate with cellular ferroptosis. On the other hand, Hmox1 is central to cellular iron metabolism and antioxidative actions [31]. We observed that fluctuations in Hmox1 expression in VILI tightly correlated with lung ferroptosis dynamics. From a mechanistic standpoint, Hmox1 may modulate ROS generation and advance ferroptosis by influencing the intracellular Fe2 + concentration [32].Cumulatively, all these genes intertwine with inflammatory reactions or oxidative stress, mirroring VILI's attributes. Assessing them collectively hints at an intricate VILI regulatory network encompassing inflammation, oxidative stress and ferroptosis. Given their interdependence, upcoming therapies should consider multifaceted interventions spanning various pathways to maximize efficacy.

The ROC curve analysis in our study underscores the significant diagnostic potential of VFHGs. When juxtaposed with existing biomarkers, VFHGs appear to boast superior specificity and sensitivity. However, the roles these genes play in VILI and how they intricately interact with ferroptosis have not been thoroughly delineated. Given this, there is a compelling need for future studies to delve into the functional dynamics of gene-miRNA networks and other intersecting pathways with ferroptosis in VILI. For validation, we used two primary methodologies: the VILI mouse model and external dataset validation. Both approaches bolster the credibility of our outcomes, underscoring the robustness and scientific integrity of our research.

Based on reports, ferroptosis and VILI are closely associated [10]. However, the relationship between ferroptosis and immunity remains unclear. Utilizing the CIBERSORT package, we investigated the alterations in the proportion of immune cells during the VILI process. The results revealed a decrease in CD8 T cells and activated NK cells, alongside an increase in gamma delta T cells, M0 Macrophages, activated mast cells and eosinophils, when compared to normal lung tissue. Understanding the specific shifts in immune cells during VILI is pivotal for guiding subsequent treatments targeting the ferroptosis pathway.

However, it is essential to acknowledge certain limitations. The databases employed could harbor inherent biases and discrepancies might exist between the mouse VILI model and human clinical scenarios. Such differences necessitate additional empirical studies to pinpoint the precise roles of these genes in VILI.

## Conclusions

To conclude, our research illuminates not only the potential association of VFHGs with VILI but also their pivotal role in ferroptosis. These findings lay a firm theoretical foundation for deepening our grasp of VILI's pathophysiological intricacies and charting new therapeutic avenues for patients afflicted with VILI.

### Supplementary Information

**Additional file 1: Supplementary Figure 1A.** PCA diagram of CON and VILI groups. (A-G) PCA diagrams of GSE9314, GSE9368, GSE11434, GSE29920, GSE2368, GSE121550 male, GSE121550 female divided into CON group and VILI group. (H) Batch-corrected combined PCA plots for different data sets.

**Additional file 2: Supplementary Figure 1B.** Boxplot after batch effect correction.

**Additional file 3: Supplementary Table 1.** Detailed methods for transmission electron microscopy and HE staining.

**Additional file 4: Supplementary Table 2.** The detailed results of BP, CC, MF and KEGG pathways.

## Data Availability

All pertinent data derived or scrutinized throughout the course of this study are comprehensively incorporated within the body of this article.Gene expression data (GSE9314, GSE9368, GSE11434, GSE29920, GSE2368, GSE121550 and GSE86229) was downloaded from the GEO and the datasets analyzed during the current study are available in the GEO repository [https://www.ncbi.nlm.nih.gov/geo].
