# Supplementary figures and images for: Delineation and authentication of ferroptosis genes in ventilator-induced lung injury
Source: BMC Med Genomics. 2024 Jan 23;17:31. doi: 10.1186/s12920-024-01804-y (PMC10804751; doi:10.1186/s12920-024-01804-y)

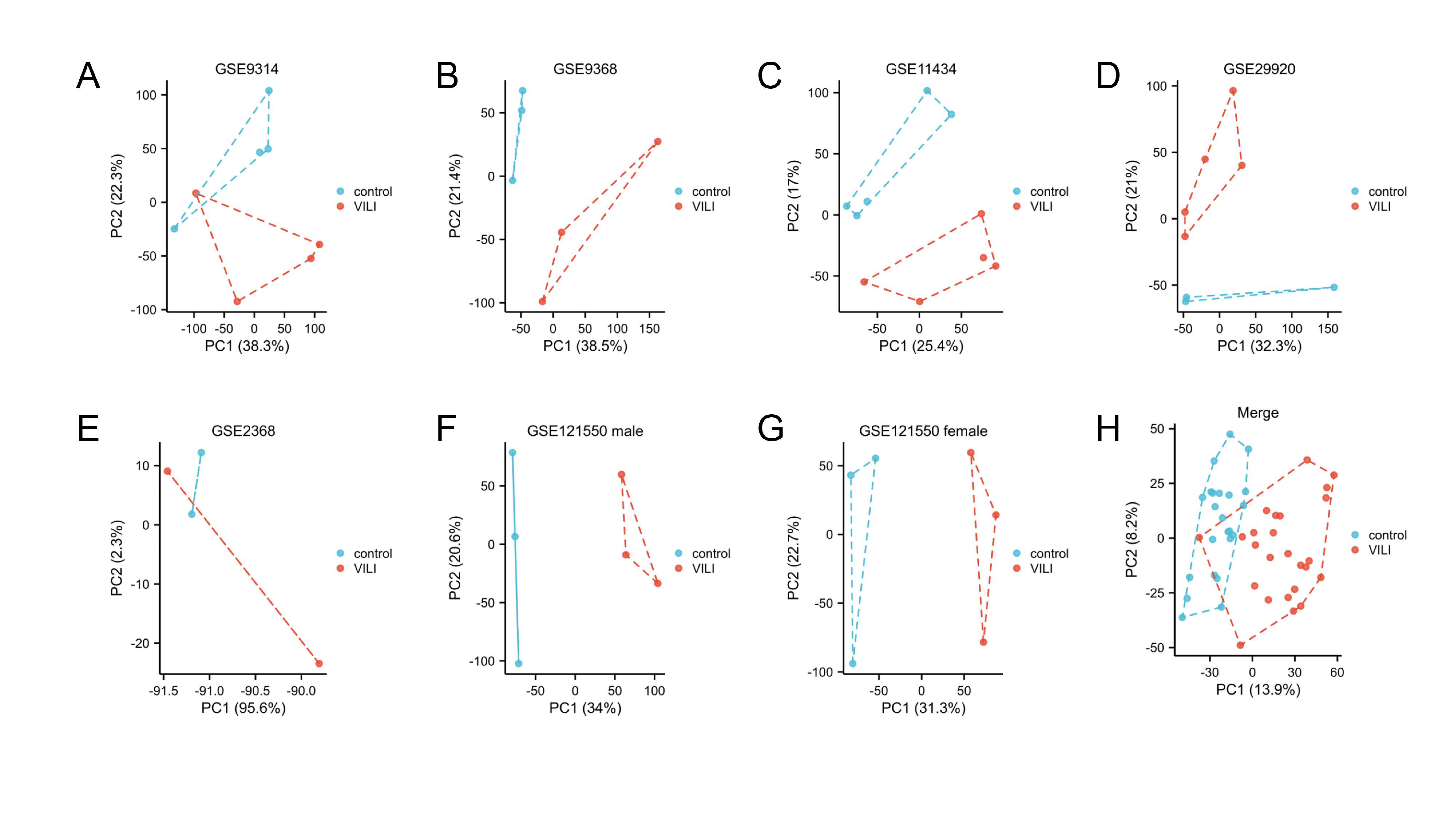

Supplement: Supplementary file 1 — Additional file 1: Supplementary Figure 1A. PCA diagram of CON and VILI groups. (A-G) PCA diagrams of GSE9314, GSE9368, GSE11434, GSE29920, GSE2368, GSE121550 male, GSE121550 female divided into CON group and VILI group. (H) Batch-corrected combined PCA plots for different data sets. [file 12920_2024_1804_MOESM1_ESM.tif]

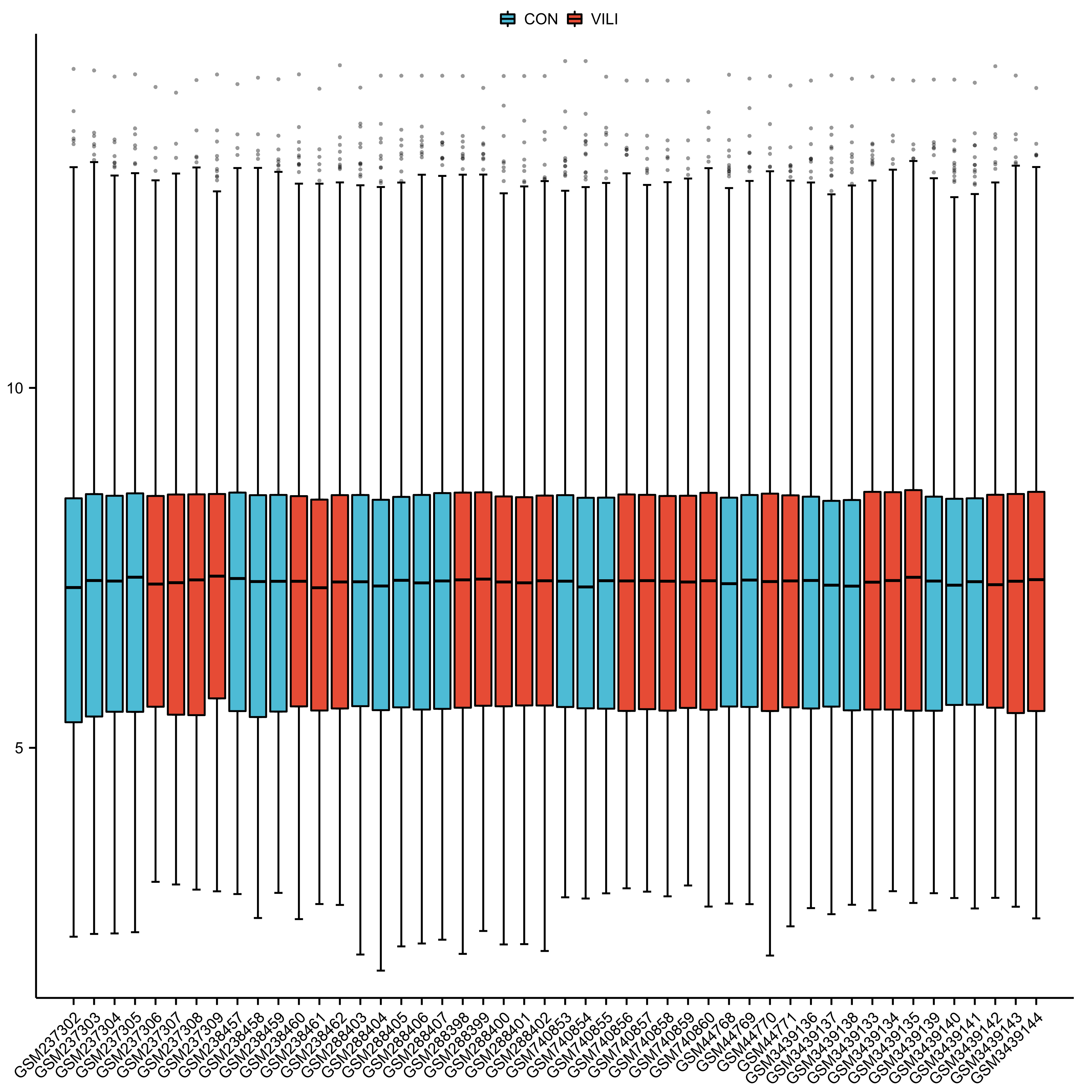

Supplement: Supplementary file 2 — Additional file 2: Supplementary Figure 1B. Boxplot after batch effect correction. [file 12920_2024_1804_MOESM2_ESM.tif]
